# Genetic factors for differentiated thyroid cancer in French Polynesia: new candidate loci

**DOI:** 10.1093/pcmedi/pbad015

**Published:** 2023-06-13

**Authors:** Monia Zidane, Marc Haber, Thérèse Truong, Frédérique Rachédi, Catherine Ory, Sylvie Chevillard, Hélène Blanché, Robert Olaso, Anne Boland, Éric Conte, Mojgan Karimi, Yan Ren, Constance Xhaard, Vincent Souchard, Jacques Gardon, Marc Taquet, André Bouville, Jean-François Deleuze, Vladimir Drozdovitch, Florent de Vathaire, Jean-Baptiste Cazier

**Affiliations:** University Paris-Saclay, UVSQ, Inserm, Gustave Roussy, CESP, Team "Radiations Epidemiology", Villejuif 94805, France; Centre for Computational Biology, University of Birmingham, Birmingham B152TT, UK; Institute of Cancer and Genomic Sciences, University of Birmingham, Birmingham B152TT, UK; University Paris-Saclay, UVSQ, Inserm, Gustave Roussy, CESP, Team "Exposome and Heredity", Villejuif 94805, France; Endocrinology Unit, Territorial Hospital Taaone, F-98713, Papeete, Tahiti 98713, French Polynesia; CEA, Laboratoire de Cancérologie Fondamentale, Institut de Biologie François Jacob, iRCM, SREIT, Laboratoire de Cancérologie Expérimentale (LCE), Université Paris-Saclay, Fontenay aux Roses 92265, France; CEA, Laboratoire de Cancérologie Fondamentale, Institut de Biologie François Jacob, iRCM, SREIT, Laboratoire de Cancérologie Expérimentale (LCE), Université Paris-Saclay, Fontenay aux Roses 92265, France; Fondation Jean Dausset-Centre d'Etude du Polymorphisme Humain, Paris 75010, France; Université Paris-Saclay, CEA, Centre National de Recherche en Génomique Humaine, Evry 91057, France; Université Paris-Saclay, CEA, Centre National de Recherche en Génomique Humaine, Evry 91057, France; U.S.R. 2003 (CNRS / UPF), Faa'a, Tahiti 98702, France; University Paris-Saclay, UVSQ, Inserm, Gustave Roussy, CESP, Team "Exposome and Heredity", Villejuif 94805, France; University Paris-Saclay, UVSQ, Inserm, Gustave Roussy, CESP, Team "Radiations Epidemiology", Villejuif 94805, France; University of Lorraine, INSERM CIC 1433, Nancy CHRU, INSERM U1116, Nancy 54500, France; University Paris-Saclay, UVSQ, Inserm, Gustave Roussy, CESP, Team "Radiations Epidemiology", Villejuif 94805, France; Hydrosciences Montpellier, Research Institute for Development, CNRS, University of Montpellier, Montpellier 62307, France; Research Institute for Development, Center IRD on Tahiti, Arue, Tahiti 98713, French Polynesia; National Cancer Institute (retired), Bethesda, MD 20892, USA; Fondation Jean Dausset-Centre d'Etude du Polymorphisme Humain, Paris 75010, France; Université Paris-Saclay, CEA, Centre National de Recherche en Génomique Humaine, Evry 91057, France; Division of Cancer Epidemiology and Genetics, National Cancer Institute, NIH, DHHS, Bethesda, MD 20892, USA; University Paris-Saclay, UVSQ, Inserm, Gustave Roussy, CESP, Team "Radiations Epidemiology", Villejuif 94805, France; Centre for Computational Biology, University of Birmingham, Birmingham B152TT, UK; Institute of Cancer and Genomic Sciences, University of Birmingham, Birmingham B152TT, UK

**Keywords:** differentiated thyroid cancer, population genetics, genetic susceptibility

## Abstract

**Background:**

Populations of French Polynesia (FP), where France performed atmospheric tests between 1966 and 1974, experience a high incidence of differentiated thyroid cancer (DTC). However, up to now, no sufficiently large study of DTC genetic factors in this population has been performed to reach definitive conclusion. This research aimed to analyze the genetic factors of DTC risk among the native FP populations.

**Methods:**

We analyzed more than 300 000 single nucleotide polymorphisms (SNPs) genotyped in 283 DTC cases and 418 matched controls born in FP, most being younger than 15 years old at the time of the first nuclear tests. We analyzed the genetic profile of our cohort to identify population subgroups. We then completed a genome-wide analysis study on the whole population.

**Results:**

We identified a specific genetic structure in the FP population reflecting admixture from Asian and European populations. We identified three regions associated with increased DTC risk at 6q24.3, 10p12.2, and 17q21.32. The lead SNPs at these loci showed respective p-values of 1.66 × 10^−7^, 2.39 × 10^−7,^ and 7.19 × 10^−7^ and corresponding odds ratios of 2.02, 1.89, and 2.37.

**Conclusion:**

Our study results suggest a role of the loci 6q24.3, 10p12.2 and 17q21.32 in DTC risk. However, a whole genome sequencing approach would be better suited to characterize these factors than genotyping with microarray chip designed for the Caucasian population. Moreover, the functional impact of these three new loci needs to be further explored and validated.

## Introduction

French Polynesia (FP) is a French Overseas Territory, only populated by the Māori people from Western Polynesia more than 1000 years ago,^1^ up to the first contact with the Europeans, between the 16th and 18th centuries, depending on the archipelago. About 93% of the population born in FP still defined themselves as Māori or of mixed Māori origin in the 1988 census, the last census to record ethnic information.^2^

From 1966 to 1974, France performed 44 atmospheric nuclear tests in the atolls of Mururoa and Fangataufa, in the southeastern part of FP, near the Gambier Islands.^3^ Although only about 500 inhabitants permanently lived less than 500 km from nuclear sites, radioactive fallouts of several tests were observed in more distant islands and atolls, including Tahiti, where more than half of population of FP lived.^4^

Since the beginning of the 2000s, we conducted a population-based case-control study on differentiated thyroid cancer (DTC) risk factors in FP. This study was prompted by the high incidence of DTC recorded in the Polynesian islands, including FP,^5–7^ and the known risks of radiation-induced thyroid health effects associated with radioactive fallout from nuclear tests.^8^ We have shown that lifestyle-related risk factors such as obesity and a high number of pregnancies are associated with DTC risk in European populations and could explain part of the high incidence of DTC in FP, due to their high prevalence in this territory.^9–11^ A family history of thyroid cancer or drinking spring water was also shown to be associated with DTC risk,^12^, ^13^ whereas a traditional Polynesian diet largely based on local vegetables and sea products was associated with a lower thyroid cancer risk.^10^ Lastly, it was also suggested that there was a small, but significant, role of nuclear tests fallouts in the incidence of DTC in FP.^6^, ^14^

In order to investigate the potential role of genetic susceptibility in the high thyroid cancer incidence observed in FP, we previously performed a candidate gene-based analysis to confirm in the French Polynesian population the role of 5 variants in 2q35, 9q22.33, 8p12 and 14q13.3 by Maillard *et al*.^11^ that were identified in former GWAS in European populations.^13^, ^15^ We reported associations of similar magnitude to what was observed in the European population but showed that the at-risk alleles of such variants were not more frequent in the French Polynesian population than in the European Population.^11^ Maillard *et al*. performed an analysis on common variants at 9q22.33, 14q13.3, and *ATM* loci and DTC in the initial case-control study included in the present analysis (about 50% of the population of the present study).^11^ The findings of this previous study highlighted an interaction between rs944289 in *NKX-1* (14q13.3) and the radiation dose to the thyroid with a higher risk for the genotype “T/T” carriers. In addition, an association was found between increased DTC risk and rs965513 near *FOXE1*.^11^ More detailed results were reported through a multiethnic GWAS and a fine-mapping analysis that included the Melanesian population from New Caledonia and only a part of the Polynesian population included in the present article.^16^, ^17^

Indeed, we extended the previous case-control study conducted in FP by including thyroid cases diagnosed from 2004 to 2016. It resulted in a confirmation of our previous results, although, slightly lowering the estimate of the risk associated with radiation exposure.^18^

In this report, we performed the first GWAS on DTC for individuals exclusively from FP to identify genetic DTC risk factors specific to this high-risk population.

## Materials and method

### Population

Cases were collected from the Cancer Incidence Registry of FP, private endocrinologists and clinics, as well as public hospitals. All patients diagnosed in 1981–2016 with DTC at the age of 55 years or less, born and resident in FP at the time of diagnosis, were eligible. Controls were healthy individuals, selected from the native population using the registry of births that covers the whole territory. Cases and controls were matched on birth date (around 3 months of maximum difference) and sex. Full details about the first study are described elsewhere.^18^, ^19^

All participants answered a detailed questionnaire about education (available as supplementary data), occupation, weight history, personal and familial history of thyroid diseases and cancers, gynecologic and reproductive history, and diet at the time of the interview and during childhood. Pathological and histological thyroid cancer details were also gathered from clinical records for each case with saliva DNA samples (Oragene^®^ kit). The study has been approved by the “Commission Nationale de l'Informatique et des Libertés” (CNIL agreements 996 007 and 900 124) and the Ethical Committee of French Polynesia. Each participant signed an individual written consent.

### Estimation of thyroid radiation dose

The methodology and results of the dose estimation process have already been described in former published studies. Thyroid radiation doses due to (i) intakes of 131I and of short-lived radioiodine isotopes (132I, 133I, 135I) and 132Te via inhalation and ingestion of foodstuffs and drinking water, (ii) external irradiation from gamma-emitting radionuclides deposited on the ground, and (iii) ingestion of long-lived 137Cs with foodstuffs were reconstructed for each study subject, mainly by using:

- Results of radiation monitoring of the environment and foodstuffs performed by the SMSR (Service Mixte de Sécurité Radiologique) and the SMCB (Service Mixte de Contrôle Biologique), the internal radiation protection services of French Army and CEA after each test, which were declassified in 2013, instead of syntheses sent by France to UNSCEAR, as in the initial study. These reports made it possible to conduct a comprehensive estimation of the ground deposition of 33 radionuclides at the time of arrival of fallout after each test, based on measurements of total ground deposition, measurements of total beta-concentration in air, or measurements of exposure rate at different locations in FP.^20^
- Integrating historical data on population lifestyle related to the period of the tests, which were collected in 2016–2017 using focus-group discussions and key informant interviews monitored in each of the 5 archipelagos of FP, in addition to declarations reported in self-questionnaires.^14^

This dosimetric reconstruction led to an estimation of the mean lifetime thyroid dose among the study subjects of around 4.7 mGy.^21^ Doses from131I intake ranged up to 27 mGy, while those from intake of short-lived iodine isotopes (132I, 133I, 135I) and 132Te ranged up to 14 mGy. Thyroid doses from external exposure ranged up to 6 mGy, and those from internal exposure due to 137Cs ingestion did not exceed 1 mGy. Intake of 131I was the main pathway of thyroid exposure accounting for 72% of the total dose.^22^

### Genotyping data

DNA was extracted from saliva samples at the CEPH-Biobank (Centre d'Etude du Polymorphism Humain, Paris, France) using an automated salt precipitation method on an Autopure (Qiagen). Genotyping was performed with a custom-made Infinium OncoArray-500K BeadChip EPITHYR (Illumina®) at the Centre National de Recherche en Génomique Humaine (CNRGH/CEA, Evry, France), on an Illumina high-throughput genotyping platform automated according to manufacturer's instructions. The OncoArray-500K is described in detail in Amos *et al*. 2017^23^ and contains 499 170 SNPs. An additional 13 759 custom markers were incorporated, based on prior evidence of association with relevant biological pathways for thyroid cancer.^16^

In total, 289 cases and 418 controls with a sufficient amount of DNA, from the first study and the extension, were genotyped. A threshold of 5% was applied for missing call rate per SNP and 10% per individual. Furthermore, three individuals were removed because they were duplicates. Hardy-Weinberg equilibrium (HWE) was also assessed per SNP among controls, using a Fisher exact test with a p-value threshold of 10^−5^. Only SNPs with minor allele frequency (MAF) above 5% were considered in the analyses to ensure sufficient power. The quality control of the genotyping data was performed with Plink2 program (version Alpha 2.3).^24^

As our genotyping array was originally designed based on the human assembly hg19, the SNPs positions were mapped to the reference genome hg38 using the Liftover tool from UCSC with default parameters^25^ (available here: https://genome.ucsc.edu/cgi-bin/hgLiftOver). The data was phased with Eagle v2.4.1^26^ according to the 1000 Genomes Project phase 3 and the hg38 assembly as references, with a maximum number of conditioning haplotypes fixed at 10 000. Then imputation was performed with IMPUTE2^27^, ^28^ for hits regions on chromosomes 6, 10, and 17 using the 1000 Genomes Project phase 3 (accessed December 2019)^29^ and the Human Genome Diversity Project (accessed December 2019)^30^ datasets as reference panels. We imputed the region 500kb on both sides of the most significant SNP position for each of the three loci of interest. To avoid strand issues, we excluded palindromic SNPs. After imputation, SNPs with an info score < 0.85 were excluded.

### Statistical methods

#### Global Admixture analyses

To study genetic structure in our population we merged our data with published data from worldwide populations previously compiled and available from https://reichdata.hms.harvard.edu/amh_repo/(v29.1). We filtered populations in the dataset keeping: Africans (Yoruba), East-Asians (Chinese, Vietnamese, and Japanese), Central and South Asians (Balochi, Brahui, and Sindhi), North-Eastern Asians (Yakut and Yukagir), other Oceanian populations (Samoan and Papuan), Metropolitan French and ancient South Pacific individuals including three ancient French Polynesians who lived during the eighteenth-century AD.^31^ Finally, data from 408 persons from the reference dataset and 56 316 shared SNPs were analyzed.

We first performed a principal component analysis (PCA) using the “smartpca” function from Eigensoft v7.2^32^ on the study population alone and found a strong genetic structure (Supplementary Fig. 1). The first 5 principal components explained 90% of the variance in our dataset. We then ran a PCA including worldwide diversity populations and projected the French Polynesians into the defined space. Based on the results from the PCA, we focused our analysis on three ancestries found in French Polynesia today: local Polynesian ancestry represented by ancient French Polynesians, East Asian ancestry represented by modern Chinese Han, and European ancestry represented by modern French. We performed an analysis using the software ADMIXTURE^33^ supervised by using only three reference populations, estimating the fraction of these different ancestries in our dataset.

#### Association analyses

Logistic mixed effects models implemented in the GENESIS R Bioconductor package were used to test for association between genotype and DTC risk, using a penalized quasi-likelihood approximation to the generalized linear mixed model. To consider the population structure, GENESIS estimates a kinship matrix that accounts for cryptic and known relatedness between subjects.^34^ This kinship matrix was derived from a set of LD-pruned genotyped SNPs (at threshold R² = 0.1) and was included as a random effect in the logistic mixed model. The three first principal components corresponding to our three founding populations were included as fixed-effect covariates. The other covariables included in the model are age (continuous), sex (binary), thyroid cancer family history (binary), goiter history (binary), radiotherapy history (binary), Mururoa site workers (binary), BMI (continuous), height (continuous), educational level (primary school, secondary school), smoking status (binary), among women the number of pregnancies longer than 7 months (0, 1–3, >3), dietary selenium and iodine intake during adulthood and childhood (continuous) and the internal radiation dose delivered to the thyroid gland before the age of 15 (continuous). The same model was also tested after the exclusion of the cases with non-invasive microcarcinoma (< 10 mm, n = 173), and with an interaction term between the estimated dose delivered to the thyroid gland before the age of 15 and the genotype.

Besides, in an attempt to replicate former results from Maillard *et al*.,^11^ we used an unconditional logistic model adjusted on the same variables as above. Benjamin Hochberg FDR procedure was used for multiple testing correction^35^ for the GWAS analysis, to better take into account the non-independence of the SNPs.

#### Local ancestry Inference using RFMix

Local ancestry inference, that infers the regional ancestral origin of a chromosomal segment in admixed populations, was performed using RFMix version 2.03,^36^ with default parameters. Then, the RFMix local ancestry calls were converted to Plink TPED files, where the encoded dosage is defined as having 0, 1, or 2 copies for each of the ancestry populations at a particular local ancestry segment.

#### Admixture mapping

The main aim of this analysis is to test for association between the local ancestry at a genomic region and DTC. These tests were performed using the linear mixed-effect models implemented in the EMMAX software package. These models included local ancestry count as the main effect and a Balding-Nichols kinship matrix as a random effect. This step was run for each of the three identified ancestries: European, South Asian, and East Asian. We adapted the correction for multiple testing from Gao *et al*.,^37^ to be used in the estimation of the number of effective tests in a Bonferroni correction for multiple testing.^38^ This number of effective tests is the sum of PC explaining more than 99.5% of genetic variance per chromosome. The significance threshold for our admixture mapping analysis is 1.13 × 10^−4^ based on 442 effective tests. This approach has already been adopted in a similar study.^39^

#### Single-SNP heritability and posthoc power calculation

Based on the summary results from the association analysis we estimated the heritability and the post-hoc power for each tested SNP. The heritability was calculated as the proportion of phenotypic variance explained by a given SNP. The following formula was used to assess single-SNP heritability h² from genome-wide association study results, where *i* is a given polymorphism:^40^


$$
\begin{equation*}
h^{2}_{ i}=\frac{2(\beta _{ i}){\rm MAF}_{ i}\,(1-{\rm MAF}_{ i})}{2(\beta _{ i})\, {\rm MAF}_{ i}(1-{\rm MAF}_{ i})+2{\rm \mathit{ N}}\,(\beta _{ i}.{\rm Se}^{2})\,{\rm MAF}_{ i}(1-{\rm MAF}_{ i})}
\end{equation*}
$$

where MAF*_i_* is the minor allele frequency for the SNP*_i_*, and allelic causal effect size is ${{\rm \beta }}$*_i_*, ${{\rm \beta }}$*_i_.Se* is the standard error of *β_i_* and *N* is the population size. Then, based on h² values, we estimated the observed power (post-hoc power) for each tested SNP*_i_*:


$$
\begin{equation*}
{\rm{Observed\ powe}}{{\rm{r}}_{\rm{i}}}{\rm{ = 1 - \mathit{ G}}}\left( {{\rm{\mathit{ t}, \lambda, 1}}} \right)
\end{equation*}
$$

where G is the cumulative distribution function of non-central Chi-square distribution, and the non-centrality parameter (λ) depends on sample size (N) and the proportion of phenotypic variance that is explained by the SNP_i_ and calculated above (h²_i_):


$$
\begin{equation*}
{\rm{\lambda }} = \left( {\frac{{{h^2}i}}{{1 - {h^2}i}}{\rm{\,\,}}} \right)N{\rm{\,\,}}
\end{equation*}
$$

Two different values of significance threshold α were considered: α_1 _= 0.05/total number of tested SNPs = 1.66 × 10^−7^ and α_2 _= 0.05/number of independent SNPs tested (D’ < 0.7 in windows of 1000 SNPs) = 1.11 × 10^−5^.

## Results

### Demographic and epidemiologic characteristics

At the end of quality control of the genotyping data, the present study included 283 cases and 418 controls and 300 908 SNPs were available for the analysis (study flowchart in Supplementary Fig. 1). Main characteristics of all cases and controls are described in Table 1: more than 80% of cases were women. The mean age of the thyroid cancer diagnosis was 44 years, and 56% of the cases were smokers. Anthropometric factors (BMI and body height) and dietary intake of selenium and iodine during childhood were found to significantly differ between cases and controls. The distributions of the thyroid doses for the cases and for the controls are presented in the Supplementary Fig. 2.

**Table 1.** Demographic and epidemiologic description.

	Controls	DTC Cases	P-value*
	(N = 418)	(N = 283)	
**Sex(%)**			
Men	58 (13.7%)	51 (18.0%)	0.1
Women	360 (86.3%)	232 (82.0%)	
**Smoker (%)**			
No	213 (50.8%)	125 (44.2%)	0.1
Yes	205 (49.2%)	158 (55.8%)	
**Height (meters)**			
Mean (SD)	1.66 (0.0762)	1.66 (0.0771)	0.6
Median [Min, Max]	1.66 [1.44, 2.01]	1.66 [1.40, 1.90]	
**Personal history of radiotherapy (%)**			
No	407 (97.4%)	265 (93.6%)	0.02
Yes	11 (2.6%)	18 (6.4%)	
**Diploma**			
No	138 (32.6%)	104 (36.7%)	0.3
Yes	281 (67.4%)	179 (63.3%)	
**Age at diagnosis in years (%)**			
1–19	19 (4.3%)	3 (1.1%)	<0.001
20–29	65 (15.6%)	20 (7.1%)	
30–39	103 (24.7%)	76 (26.9%)	
40–49	145 (34.8%)	37 (13.1%)	
50–59	86 (20.6%)	147 (51.9%)	
**BMI (kg/m²)**			
Mean (SD)	28.3 (6.90)	31.4 (7.38)	<0.001
Median [Min, Max]	27.1 [16.0, 56.5]	30.7 [17.1, 66.1]	
**Dietary iodine intake during childhood (µg/day)**			
Mean (SD)	125 (86.7)	150 (130)	0.005
Median [Min, Max]	107 [0, 841]	116 [0, 934]	
**Dietary selenium intake during childhood (µg/day)**			
Mean (SD)	109 (74.8)	138 (151)	0.003
Median [Min, Max]	92.8 [0, 432]	99.1 [0, 1080]	
**Radiation dose at thyroid gland before the age of 15 (mGy)**			
Mean (SD)	1.48 (2.54)	1.39 (2.69)	0.7
Median [Min, Max]	0 [0, 13.0]	0 [0, 22.0]	

* Univariate logistic regression

### Population structure

**Figure 1.**
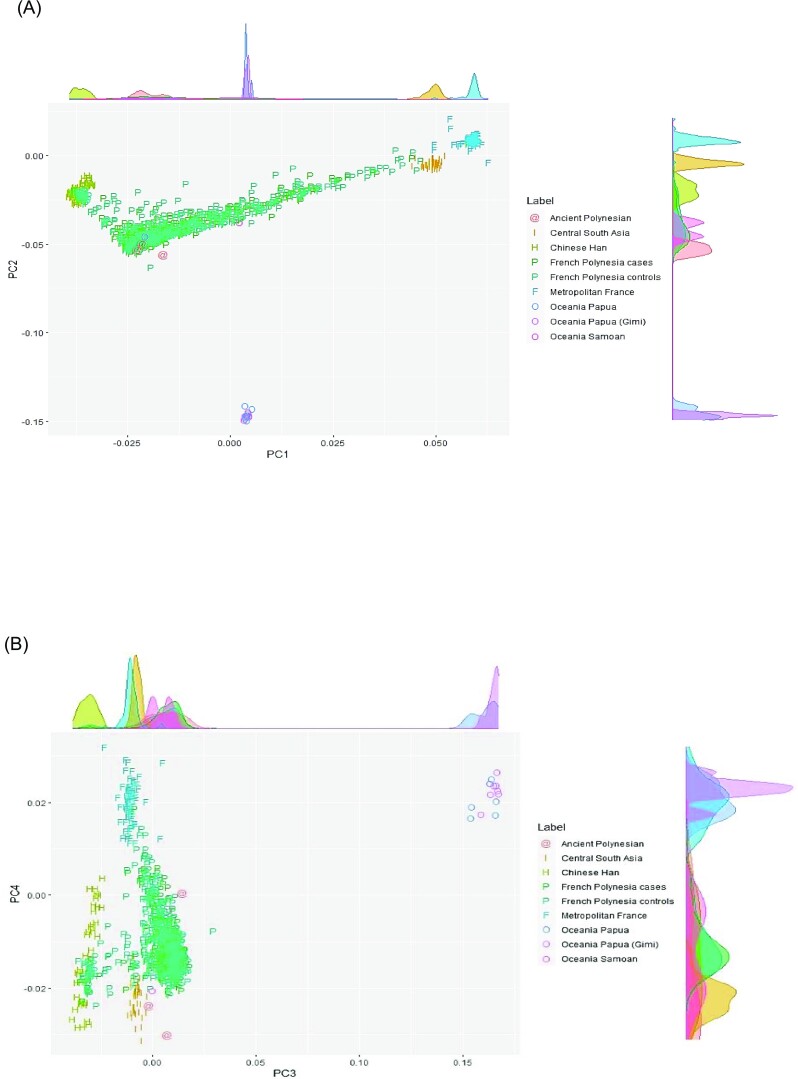
First four principal components with the corresponding proportion of explained variance in a worldwide PCA: (A) PCs 1 & 2; (B) PCs 3 & 4. We show a subset of the PCA with French Polynesians in a regional context.

**Figure 2.**
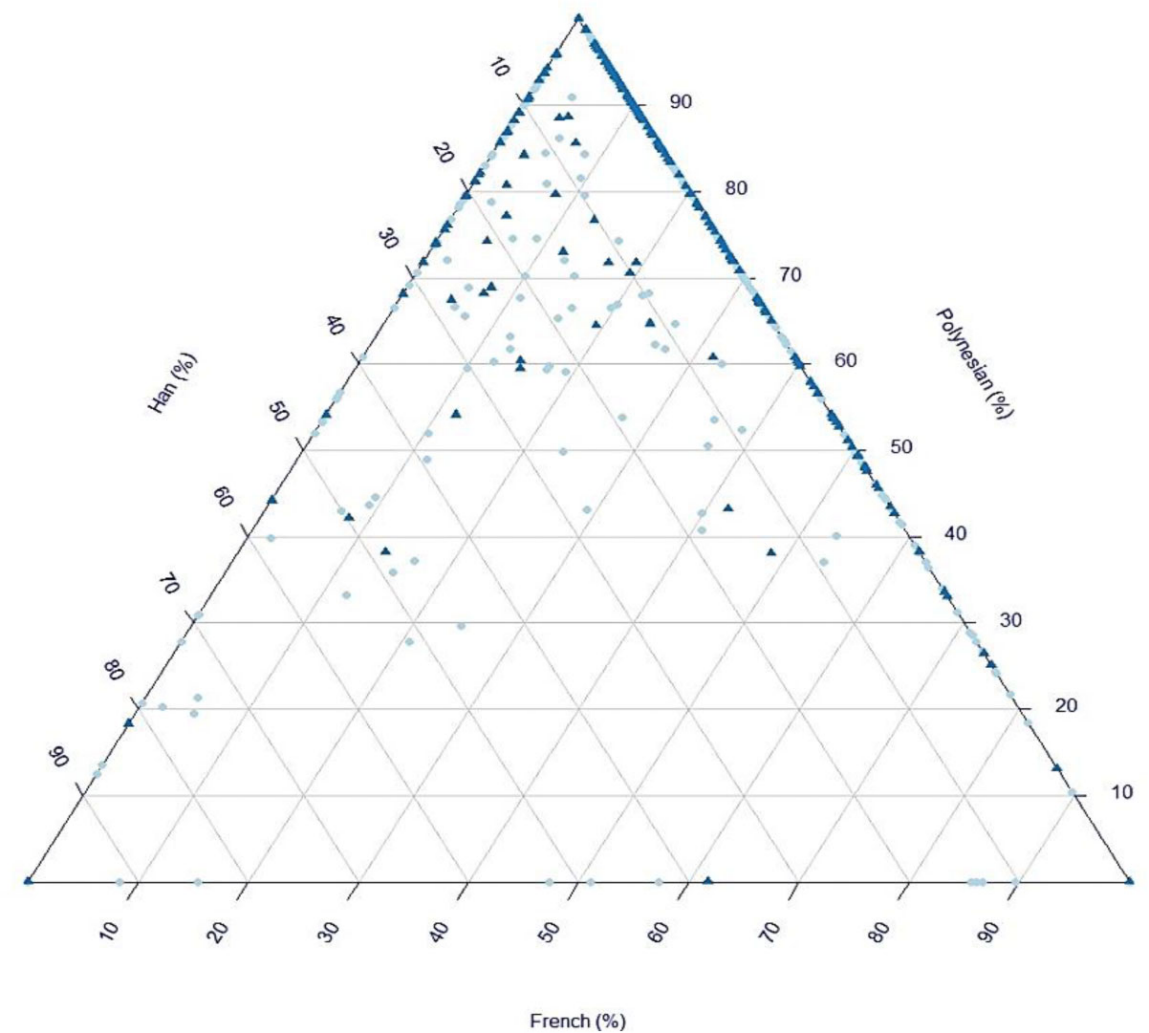
ADMIXTURE analysis showing ancestry percentage of individuals in our dataset. The three corners correspond to French, Han and ancient Polynesians used as reference groups. Cases are presented by dark blue triangles and controls by light blue dots.

PCA of the French Polynesian population revealed a significant genetic structure in the population (Supplementary Fig. 3). When the French Polynesian population was projected on a PCA defined by worldwide populations we identified two ancestry clines showing a group of French Polynesians that were drawn towards East Asians compared to the position of ancient Polynesians while a second group was drawn towards Europeans (Fig. 1A & B). Our admixture test confirmed East Asian-related and European-related admixture in French Polynesia (Fig. 2, Supplementary Fig. 4). PCA results including African and other Asian ethnicities are not shown.

### Association and admixture mapping results

**Figure 3.**
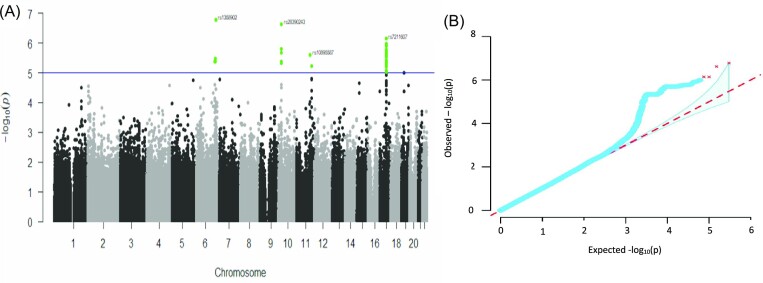
GWAS results. (A) Manhattan plot of the 300 908 SNPs (represented underneath) for DTC risk. Color represents the various chromosomes; the blue dotted line indicates p-value < 10^-5^. (B) The blue line represents the observed -log_10_p-values; the red dotted line represents the expected -log_10_p-values; the blue area represents the 95% confidence interval. The genomic inflation factor λ = 1.03.

The GWAS allowed us to identify 3 loci as highlighted in the Manhattan plot presented in Fig. 3A. The genomic inflation factor λ was = 1.03, confirming a lack of systematic genetic bias between our cases and controls (Fig. 3B). The lowest p-value was observed for rs1358902 (1.66 × 10^−7^; FDR = 0.039; OR_allelic _= 2.01) located at 6q24.3, in an intergenic region (tested allele frequency = 0.38, genotypic count detailed in Supplementary Table 1). At 10p12.2, rs28390243 had a p-value = 2.39 × 10^−7^ (FDR = 0.026), with an OR_allelic _= 1.89 (tested allele frequency = 0.38, genotypic count detailed in Supplementary Table 1). All the SNPs associated to DTC at 10p12.2 were near or within the *PIP4K2A* gene region.

A third susceptibility loci was observed at 17q21.32, which contain a large linkage disequilibrium region, mostly in the *SKAP1* gene. The minor allele of the lead SNP rs7211607 (tested allele frequency = 0.16, genotypic count detailed in Supplementary Table 1) in the *SKAP1* region had an OR_allelic_ = 2.37, (p-value of 7.19 × 10^−7^ and FDR of 0.026). Same associated regions were found to have the lowest p-values when excluding cases with non-invasive microcarcinoma from the analyses (Results not shown). The lead best SNP in this analysis was rs28390243, but not significant (OR_allelic_ = 2.04, FDR = 0.49).

Results from local imputation in each of the three regions on chromosomes 6, 10, and 17 found that the original lead SNPs in the three regions remained the most significant.

The statistical power to detect an association with rs1358902 (6q24.3) while considering the significance threshold at α_1_ was 52% and 81% at α_2_. For rs28390243 (10p12.2), the power was 50% at α_1_ and 80% at α_2_. This power was equal to 39% and 72% respectively at α_1_ and α_2_ for rs7211607 (17q21.32)_._

The estimates of the radiation doses before the age of 15 years to the thyroid gland were low, with a maximal dose of 22 mGy among cases, and 13 mGy among controls.

In the interaction analysis, none of the SNPs reached the significance threshold. The most significant was observed in many SNPs (in LD: R² between 0.85 and 1, D’ = 1) located in 21q21.1, all in the *MIR99AHG* region. These results did not change when we excluded individuals with a radiotherapy history and when non-invasive microcarcinoma cases were excluded.

We compared our findings with results from our previous study that were based on a subpopulation the current study (137 cases and 218 controls) (Maillard *et al*.^11^). We failed to find the same significant interaction in rs944289. However, the main effect of the genotype “T/T” had the same direction as in Maillard *et al*. findings^11^ (OR_T/T genotype _= 1.45, 95% Confidence Interval (CI) = 0.26–7.98) (Allelic ORs are listed in Supplementary Table 2).

According to our significance threshold for the admixture mapping analysis, only two loci in chromosomes 14 and 15 were found to be significantly associated with DTC risk in a population of East-Asian ancestry. The locus 15q14 (34 830 119–34 903 911) was located within the *AQR* gene region, with a p-value = 2.95 × 10^−5^. The second locus at 14q21.2 (43 863 839–43 935 211), with a p-value = 4.20 × 10^−5^, was located in an intergenic region. Results from the analysis of haplotypes coded according to European ancestry showed an association with the *PIP4K2A* gene region in 10p12.2 (22 556 003–22 730 905), with a p-value = 1.11 × 10^−6^, the very same result from the global GWAS. When this analysis was run with a haplotype coding according to the South-Asian ancestry, only one locus in the *RYBP* gene region (chromosome 3:72 374 593–72 446 623) was associated with DTC risk with a p-value = 8.37 × 10^−5^. The other top associated loci were similar to those found in analysis with a haplotype coding according to European Ancestry. Results from this analysis are shown in Supplementary Table 3.

## Discussion

The main purpose of this study was to explore the role of genetic factors in DTC occurrence in the FP population. Compared to the GLOBOCAN statistics, the sex ratio in thyroid cancer cases worldwide is 3.87, which is similar to our study.^41^ Smoking habits are more frequent in our study population compared to the general population in FP (36.5% of men and 41.4% of women smokers).^42^

Our results from the GWAS suggest potential DTC susceptibility loci at 6q24.3, 10p12.2, and 17q21.32. While all these regions have been previously linked to cancer, none of them was previously associated with DTC risk.

At locus 6q24.3, all variants were in non-coding regions, the closest genes were *GRM1, RAB32*, and *ADGB*. These genes have been associated with the risk and prognosis of ovarian cancer,^43^ gastric cancer^44^ and breast cancer.^45^ In addition, this region is also associated with autoimmune thyroiditis^46^ and in the regulation of proliferation and invasion of thyroid cancer cells^47^ and with familial lung cancer risk.^48^

The lead SNP (rs1358902) in chromosome 10 is located near a promoter for the *PIP4K2A* gene in the 10p12.2 locus. This locus has been associated with childhood B-cell acute lymphoblastic leukemia occurrence by a previous GWAS,^49^ in the European ancestry population, and in multiple other populations including Hispanic, African, and Chinese.^50^, ^51^ Variants in this gene were also found to be associated with acute lymphoblastic leukemia prognosis.^52^, ^53^ The results from a cross-ancestry meta-analysis identified an association between a variant in the *PIP4K2A* gene region and breast cancer susceptibility in European and Asian populations.^54^ Although the role of *PIP4K2A* is not well-defined and mainly depends on the biological context, this gene has recently been suggested to be involved in oxidative stress, cellular senescence, and tumor growth.^55^, ^56^ It has also been suggested to play an oncogenic role and was found to be frequently overexpressed in multiple types of leukemia and some types of solid cancers in cancer cohorts including TCGA.^55^, ^56^

Another signal was identified on chromosome 17 in the region of *SKAP1* (SRC kinase-associated phosphoprotein 1 gene (Fig. 3A)) which had been previously reported to be associated with ovarian cancer in the European and Chinese Han populations.^57^, ^58^ The expression levels of these genes were found to be associated with ovarian, endometrial, and colorectal cancer risk.^59–61^ The 17q21.32 locus is additionally associated with endometrial, prostatic, and renal cancers.^62–65^ The biological function of *SKAP1* related to these cancer types is not yet well elucidated.

The observed power under the α_1_ assumption varied from 39% to 51% for the three leading SNPs of the region. This power is between 78% and 81% if we consider α_2_. In view of the small size of the general PF population (almost 300 000), the present study included an important proportion of the total DTC cases occured in FP.

In radiation-related DTC studies, only two variants in *FOXE1* and *ATM* were associated with the disease in two different populations.^66^ In *FOXE1*, only the association of rs965513 with radiation-related DTC could be replicated in a population exposed to higher levels of radiation caused by Chornobyl disaster.^67^ Notably, the same variant rs1801516 in *ATM* could have an opposite effect between the FP population^11^ and the Belarusian population.^68^ This could highlight the importance of taking into account ancestry when considering the genetic susceptibility to DTC, and the importance of further exploration of the concerned genetic regions, as the causal variant could be a different one in the same region.

We did not observe a significant interaction between thyroid radiation dose due to atmospheric nuclear tests and genetic factors, but this result was expected given the very low level of exposure resulting in a limited statistical power for testing this interaction.

The interaction found by a former study with different doses estimation and genotyping technology^11^ was not replicated in the current study. This difference could be caused by the different thyroid IR doses between both studies, the partially shared study population, and the difference between genotyping technologies, as about 10% of the genotyping results are different between the first and the second studies.

A more recent study focused on the genomic profile of radiation-related papillary thyroid cancer after the Chornobyl accident, which included normal and tumor tissues, with whole-genome, mRNA, and microRNA sequencing; DNA methylation profiling; and genotyping arrays. The germline DNA analyses yielded three possible associations with the radiation dose of variants in SMAD3, TERT, and 9q22.33.^69^ However, none of these genes/loci were found in our study. Thus, our results from genetic susceptibility analysis identified novel genes related to DTC with many of them linked to hormonal-dependent cancers. Results from admixture mapping highlighted two regions: 15q14 (34 830 119–34 903 911) and 14q21.2 (43 863 839–43 935 211) to be associated with DTC risk in a population with East Asian ancestry. These results should be taken with precaution because of the limited sample size of the study and the high admixture of this population. Altogether, results from this analysis show that the outline of the genetic regions associated with DTC risk is relatively different depending on the ethnic origins.

The significant associations identified in our study need to be replicated and functionally validated to understand the biological mechanisms involved. An increase in the sample size in the future will permit the exploration of further orders of interaction such as the interaction between gene sets.

In addition to genetic susceptibility results, our study identified significant admixture in FP related to Asian and European populations. However, the dominating ancestry in FP is related to ancient local French Polynesians. Our study suggests that our results were not biased by population structure or admixture, especially because of the use of a mixed model. Future exploration of ancestry in FP could enable the study of the potential correlation between geographic regions (archipelago and atolls), ancestry, and DTC. A larger study population could help to better understand these potential interconnections, especially comparing to classic GWAS study population sizes, though increasing the sample size may be difficult due to the small size of the French Polynesian population and therefore the relatively small number of DTC cases in this population. Besides, the use of non-ethnically adapted microarrays strongly reduced the number of analyzable SNPs and genomic regions, as these microarrays are designed for European population and doesn't cover the same variants as in FP population. Exploring genetic factors of DTC in non-European populations will allow to propose better polygenic risk scores by identifying other variants.

## Conclusion

The French Polynesian population has genetic, geographic, and cultural specificities. Here, we attempted to study genetic DTC risk factors while accounting for genetic structure in an admixed population. Our results are suggestive of a role of three loci: 6q24.3, 10p12.2, and 17q21.32. The genes present at these loci were not previously associated with DTC, but rather with other hormone-dependent cancers or psychiatric disorders. Given that the microarray used for genotyping in the present study was designed for the population of European ancestry, it would be more adapted to perform whole genome sequencing in the Polynesian population.

## Supplementary Material

pbad015_Supplemental_File
