# Supplementary material for: Genetic factors for differentiated thyroid cancer in French Polynesia: new candidate loci
Source: Precis Clin Med. 2023 Jun 13;6(2):pbad015. doi: 10.1093/pcmedi/pbad015 (PMC10294640; doi:10.1093/pcmedi/pbad015)
Supplement: pbad015_Supplemental_File [file pbad015_supplemental_file.docx]

Supplementary Table 1 First best 20 SNPs from GENESIS association analysis

| Variant | Chr^(1)^ | Position | Risk allel (A1) | Major allele (A2) | A1 frequency in cases ^(2)^ | A1A1 genotypes frequency in cases ^(3)^ | A1A2 genotypes frequency in cases ^(4)^ |  | A2A2 allele genotypes frequency in cases ^(5)^ | A1 frequency in controls ^(6)^ | A1A1 genotypes frequency in controls ^(7)^ | A1A2 genotypes frequency in controls ^(8)^ | A2A2 genotypes frequency in controls ^(9)^ | p value | Odds-ratio ^(10)^ |
| --- | --- | --- | --- | --- | --- | --- | --- | --- | --- | --- | --- | --- | --- | --- | --- |
| rs1358902 | 6 | 146507786 | G | A | 0.36 | 0.13 | 0.46 |  | 0.42 | 0.25 | 0.06 | 0.38 | 0.56 | 1.66^E^-07 | 2.02 |
| rs28390243 | 10 | 22503563 | A | C | 0.46 | 0.21 | 0.50 |  | 0.29 | 0.33 | 0.11 | 0.44 | 0.45 | 2.39^E^-07 | 1.89 |
| rs11599280 | 10 | 22504064 | A | G | 0.43 | 0.19 | 0.49 |  | 0.32 | 0.31 | 0.10 | 0.43 | 0.48 | 1.54^E^-06 | 1.83 |
| rs8081754 | 17 | 48251305 | A | G | 0.20 | 0.04 | 0.32 |  | 0.64 | 0.12 | 0.01 | 0.21 | 0.78 | 2.00^E^-06 | 2.28 |
| rs7222593 | 17 | 48253072 | C | T | 0.20 | 0.04 | 0.32 |  | 0.64 | 0.12 | 0.01 | 0.21 | 0.78 | 2.00^E^-06 | 2.28 |
| rs7211607 | 17 | 48258937 | T | C | 0.21 | 0.04 | 0.33 |  | 0.63 | 0.12 | 0.01 | 0.21 | 0.78 | 7.19^E^-07 | 2.37 |
| rs12940931 | 17 | 48259402 | C | T | 0.20 | 0.04 | 0.32 |  | 0.64 | 0.12 | 0.01 | 0.21 | 0.78 | 1.66^E^-06 | 2.30 |
| rs17621199 | 17 | 48264913 | A | G | 0.20 | 0.04 | 0.32 |  | 0.64 | 0.12 | 0.01 | 0.21 | 0.78 | 1.75^E^-06 | 2.29 |
| rs17621297 | 17 | 48269125 | G | A | 0.20 | 0.04 | 0.32 |  | 0.64 | 0.12 | 0.01 | 0.21 | 0.78 | 1.91^E^-06 | 2.29 |
| rs12948128 | 17 | 48277311 | G | A | 0.20 | 0.04 | 0.32 |  | 0.64 | 0.12 | 0.01 | 0.21 | 0.78 | 1.85^E^-06 | 2.29 |
| rs17621707 | 17 | 48281916 | C | T | 0.20 | 0.04 | 0.32 |  | 0.64 | 0.12 | 0.01 | 0.21 | 0.78 | 1.96^E^-06 | 2.28 |
| rs17694895 | 17 | 48284393 | C | G | 0.20 | 0.04 | 0.32 |  | 0.64 | 0.12 | 0.01 | 0.21 | 0.78 | 1.17^E^-06 | 2.33 |
| rs8076655 | 17 | 48286425 | C | T | 0.20 | 0.04 | 0.32 |  | 0.64 | 0.12 | 0.01 | 0.21 | 0.78 | 1.80^E^-06 | 2.29 |
| rs7206951 | 17 | 48319469 | G | A | 0.20 | 0.04 | 0.32 |  | 0.64 | 0.12 | 0.01 | 0.21 | 0.78 | 1.71^E^-06 | 2.29 |
| rs35509644 | 17 | 48340785 | A | T | 0.20 | 0.04 | 0.32 |  | 0.63 | 0.12 | 0.01 | 0.21 | 0.78 | 7.21^E^-07 | 2.39 |
| rs1553753 | 17 | 48343444 | C | T | 0.22 | 0.05 | 0.35 |  | 0.60 | 0.14 | 0.02 | 0.24 | 0.74 | 1.97^E^-06 | 2.23 |
| rs17696503 | 17 | 48354476 | G | A | 0.20 | 0.04 | 0.32 |  | 0.64 | 0.12 | 0.01 | 0.21 | 0.78 | 1.87^E^-06 | 2.29 |
| rs35759756 | 17 | 48355625 | A | G | 0.21 | 0.04 | 0.33 |  | 0.63 | 0.12 | 0.01 | 0.21 | 0.77 | 1.25^E^-06 | 2.33 |
| rs12945429 | 17 | 48379685 | C | T | 0.21 | 0.04 | 0.33 |  | 0.63 | 0.12 | 0.01 | 0.21 | 0.77 | 1.03^E^-06 | 2.36 |
| rs12939680 | 17 | 48381289 | C | T | 0.20 | 0.04 | 0.32 |  | 0.64 | 0.12 | 0.01 | 0.21 | 0.78 | 1.71^E^-06 | 2.29 |

(6) Frequency of the risk allele (A1) in controls

(7) Frequency of homozygous risk allele genotypes (A1A1) in controls

(8) Frequency of hoterozygous genotypes (A1A2) in controls

(9) Frequency of homozygous major allele genotypes (A2A2) in controls

(10) Odds Ratiocalculated with Genesis association model

1. Chromosome number
2. Frequency of the risk allele (A1) in cases
3. Frequency of homozygous risk allele genotypes (A1A1) in cases
4. Frequency of hoterozygous genotypes (A1A2) in cases
5. Frequency of homozygous major allele genotypes (A2A2) in cases

Supplementary Table 2 Best admixture mapping results according to the identified ancestry

2 a Results in EAS identified loci

| Loci | Chromosome | Start position | p value |
| --- | --- | --- | --- |
| 3-1703 | 3 | 72448183 | 8.37E-05 |
| 10-709 | 10 | 22585781 | 1.33E-04 |
| 10-710 | 10 | 22625092 | 1.33E-04 |
| 8-1177 | 8 | 34634867 | 1.58E-04 |
| 10-711 | 10 | 22679316 | 2.15E-04 |
| 2-24 | 2 | 1170298 | 2.27E-04 |
| 2-25 | 2 | 1206663 | 2.27E-04 |
| 2-26 | 2 | 1258021 | 2.27E-04 |
| 2-27 | 2 | 1321277 | 2.27E-04 |
| 1-1083 | 1 | 59140405 | 2.28E-04 |

2 b Results in EUR identified loci

| Loci | Chromosome | Start position | p value |
| --- | --- | --- | --- |
| 10-709 | 10 | 22585781 | 1.11E-06 |
| 10-710 | 10 | 22625092 | 4.79E-06 |
| 10-711 | 10 | 22679316 | 1.53E-05 |
| 4-19 | 4 | 179156799 | 2.85E-05 |
| 4-22 | 4 | 179342532 | 8.83E-05 |
| 8-39 | 8 | 100589602 | 1.28E-04 |
| 8-38 | 8 | 100528040 | 1.32E-04 |
| 8-37 | 8 | 100464642 | 1.54E-04 |
| 4-20 | 4 | 179231670 | 1.80E-04 |
| 4-21 | 4 | 179291730 | 1.91E-04 |

Supplementary Table 3 Results comparison with Maillard et al^.1^

| RA§ | CHR¤ | Current study | | | | | Maillard et al. findings (association) | | | |
| --- | --- | --- | --- | --- | --- | --- | --- | --- | --- | --- |
|  |  | RAF* | | Main effect | | | RAF* | | Main effect | |
|  |  | Cases  N=283 | Controls  N= 418 | OR  (95%CI) | p-value | Cases  N=160 | | Controls  N=248 | OR  (95%CI) | p-value |
| rs944289  Allele T | 14 |  |  |  |  |  |  |  |  |  |
|  |  | 0.32 | 0.28 | 1.37 (1.07-1.77) | 0.01 | 0.32 | | 0.27 | 1.30 (0.97–1.74) | 0.08 |
| rs965513  Allele A | 9 |  |  |  |  |  |  |  |  |  |
|  |  | 0.27 | 0.24 | 1.13 (0.87-1.47) | 0.36 | 0.27 | | 0.21 | 1.50 (1.06–2.12) | 0.02 |
| rs1867277  Allele A | 9 |  |  |  |  |  |  |  |  |  |
|  |  | 0.20 | 0.20 | 0.99 (0.74-1.32) | 0.94 | 0.2 | | 0.19 | 1.20 (0.81–1.79) | 0.4 |
| rs1801516  Allele A | 11 |  |  |  |  |  |  |  |  |  |
|  |  | 0.02 | 0.02 | 1.19 (0.52-2.74) | 0.67 | 0.03 | | 0.02 | 3.13 (1.17–8.31) | 0.02 |

§: Risk allele

¤: Chromosome

*: Minor allele frequency

Reference: 1. Maillard S, Damiola F, Clero E, *et al*. Common variants at 9q22.33. 14q13.3. and ATM loci. and risk of differentiated thyroid cancer in the French Polynesian population. Zhang Z. editor. PLoS One [Internet]. 2015 [cited 2018 Jun 18];10:e0123700. Available from :https://www.ncbi.nlm.nih.gov/pmc/articles/PMC4388539/pdf/pone.0123700.pdf

687 potential controls

- 22 dead before interview
- 64 not localised or outside of French Polynesia
- 46 Refusals

555 controls included

Genetic study

457 cases of differentiated thyroid cancer

- 21 dead before interview
- 16 not localised
- 24 refusals
- 1: too ill to be interviewed

289 cases with DNA sample

418 with DNA sample

283 cases successfully genotyped and included

418 cases successfully genotyped and included

395 cases included

Supplementary Figure 1: Study flowchart, cases are present in the left part, controls in the right part


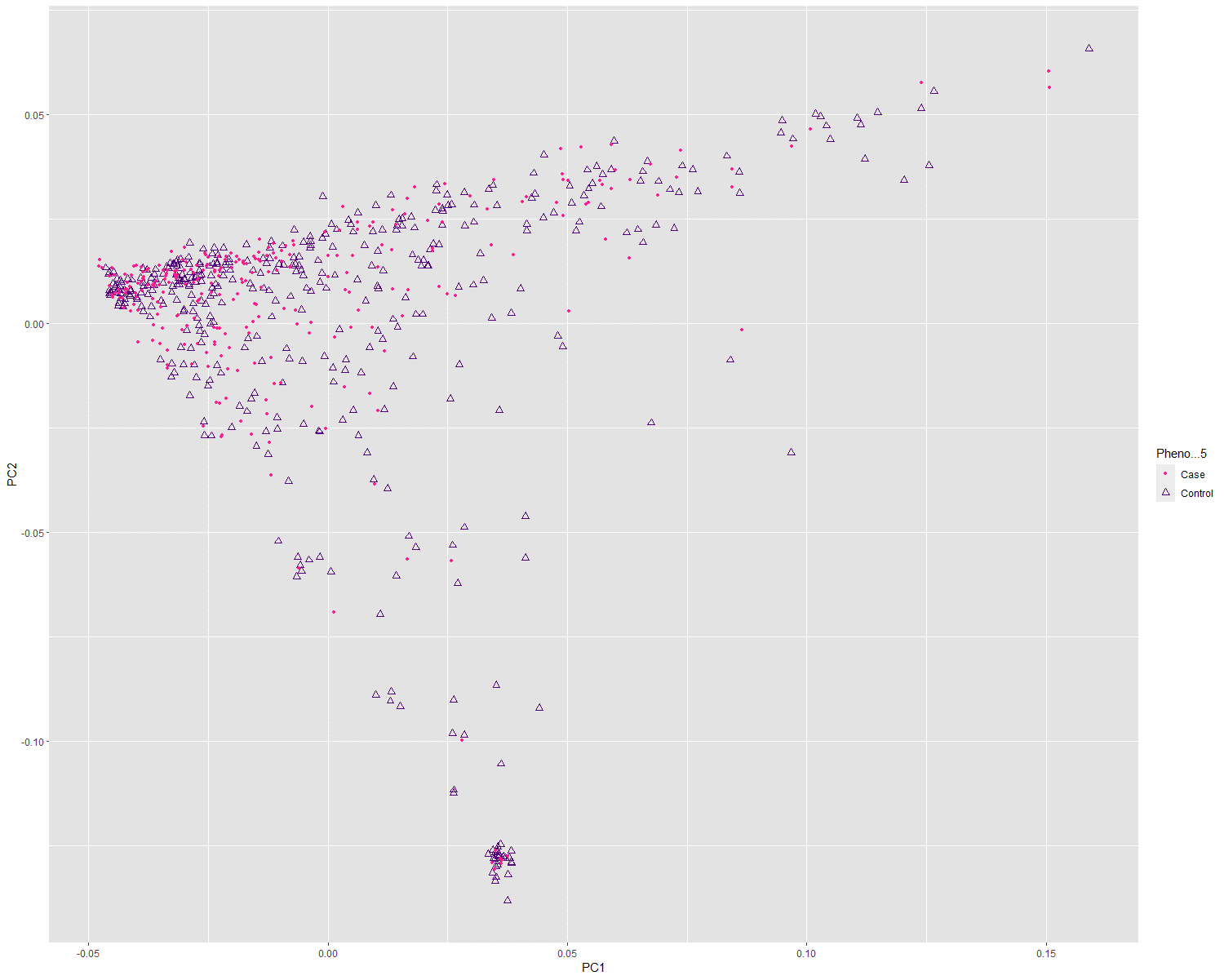


PC1

(57.03%)

Case control status

PC2 27%

Supplementary Figure 2: Four principal components with the proportions of explained variance in French Polynesian population, color as cases (pink dots) and controls (cyan triangle). The corresponding distribution of each component is projected by status on the opposite axis.


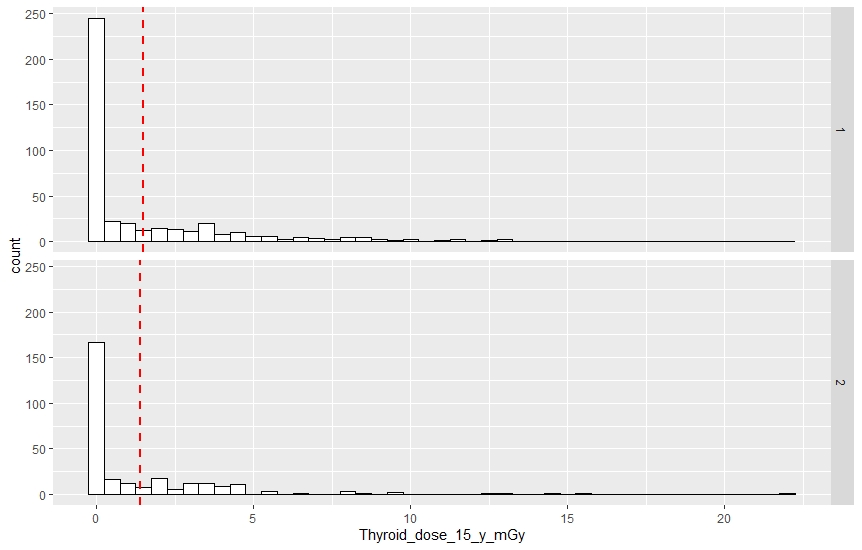


Mean=1.48

Median= 0

Mean=1.48

Median= 0

Controls

Cases

Thyroid doses in mGy

Supplementary Figure 3: Thyroid doses distribution in cases (bottom) and controls (upper). The dashed red line shows the mean value among each group.


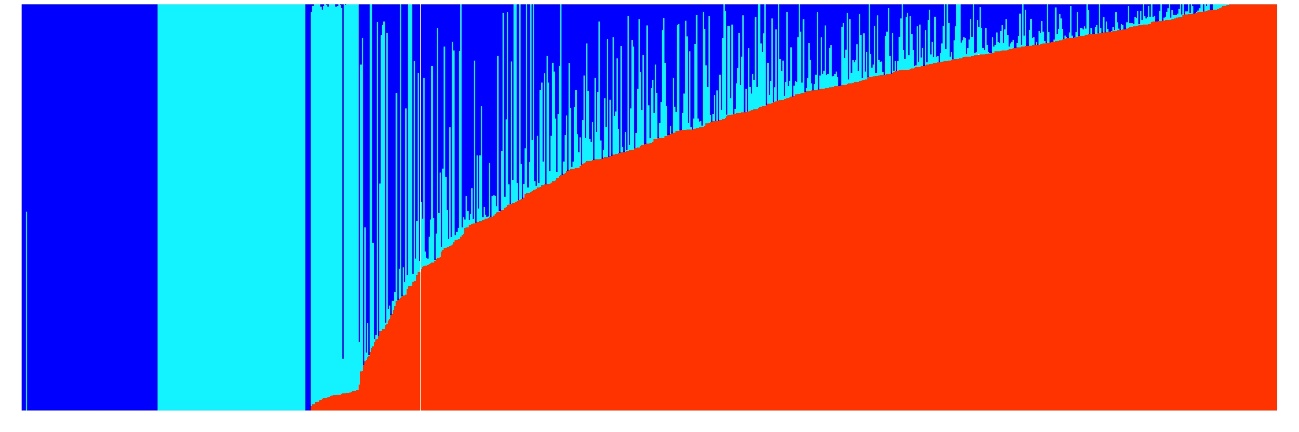


French reference population

Ancient Polynesian DNA

French Polynesian target population

Chinese Han reference population

Supplementary Figure 4: Results from supervised ADMIXTURE analysis of the studied population with three reference populations: Metropolitan French (dark blue). Chinese Han (light blue). and Ancient French Polynesians (Red). Each horizontal line represents an individual. the colors represent the proportion of each ancestry
